# Mechanism and kinetics of magnetite oxidation under hydrothermal conditions†

**DOI:** 10.1039/c9ra03234g

**Published:** 2019-10-18

**Authors:** Zimin Li, Corinne Chanéac, Gilles Berger, Sophie Delaunay, Anaïs Graff, Grégory Lefèvre

**Affiliations:** Chimie ParisTech—CNRS, Institut de Recherche de Chimie Paris, PSL Research University France gregory.lefevre@chimieparistech.psl.eu; UPMC Univ Paris 06, CNRS, Collège de France, LCMCP France; CNRS, Université Toulouse, IRAP, Sorbonne Universités France; Department of Materials and Mechanics of Components, EDF R&D France

## Abstract

The stability of magnetite under oxidizing hydrothermal conditions was evaluated at temperatures of 120, 150, 180 and 275 °C. A well-characterized sample of commercially-available magnetite with a particle size of approximately 690 nm was oxidized by dissolved oxygen (DO) under alkaline hydrothermal conditions in titanium autoclaves. In these trials, the DO was always in equilibrium with the gas phase oxygen that was air-derived and was located above the hydrothermal solution, which contained ammonium hydroxide at a pH_25 °C_ of approximately 9.5. Samples recovered by filtration were analysed by X-ray diffraction and scanning electron microscopy, while Fe(ii)/Fe ratios were determined by titration in conjunction with spectrophotometry. Oxidation between 120 and 180 °C was found to generate high concentrations of maghemite and hematite in the product, with the latter compound having either a hexagonal bipyramidal or rhombohedral morphology. The oxidation kinetics was consistent with a diffusion controlled process. The reaction probably proceeded *via* the outward diffusion of ferrous ions from the magnetite, forming a magnetite/maghemite core/shell structure in conjunction with the dissolution of maghemite and reprecipitation of hematite. Oxidation at 275 °C presented different characteristics from those observed at the lower temperatures. Negligible amounts of maghemite were found, and the primary oxidation product was hematite with no specific morphologies. Moreover, the kinetics was slower than at 180 °C. This unexpected temperature effect is attributed to the rapid growth, at 275 °C, of a dense layer of hematite on the surface of the magnetite that impeded the oxidation of magnetite.

## Abstract 2

Oxidation kinetics of magnetite into hematite depends on temperature and morphology of the ferric oxide phase.

## Graphical abstract

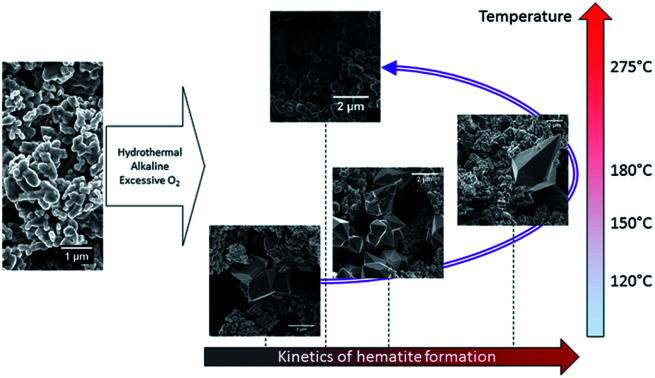

## Introduction

**Fig. 1:**
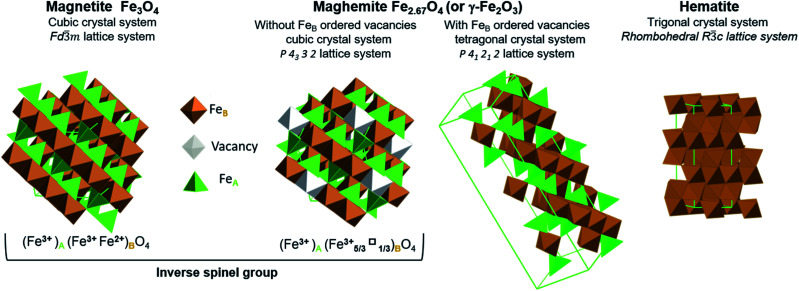
Representation of crystal structures and space group data of magnetite, maghemite (with ordered and disordered vacancy distributions) and hematite.

Iron oxides have attracted significant attention for many decades because they have numerous potential applications, including in energy production, data storage, magnetic resonance imaging, lithium storage, catalysis and ferrofluids, as well as in the fields of life, environmental and earth sciences.^1–10^ Hybrids composed of iron oxides and graphene also have numerous promising uses.^11–14^ The iron oxides that can be employed in this manner include magnetite (Fe_3_O_4_), maghemite (γ-Fe_2_O_3_) and hematite (α-Fe_2_O_3_) (Fig. 1).

Stoichiometric magnetite (Fe_3_O_4_) contains one divalent iron atom while the other two are trivalent. This compound has a face centred cubic inverse spinel structure and belongs to the space group *Fd*3̄*m*. The ferrous ions occupy half the octahedral sites, while the ferric ions are evenly situated at both octahedral and tetrahedral sites. Magnetite can be synthesized through the co-precipitation of ferric and ferrous ions. While magnetite is a mixture of iron atoms having different valence values, maghemite and hematite are both trivalent iron oxides. Maghemite has the same crystal structure as magnetite, consisting of a close-packed oxygen array, but with fewer iron atoms and more vacancies. Ferric ions occupy both tetrahedral and octahedral sites while vacancies are situated only at octahedral sites. When the distribution of vacancies in maghemite is ordered, the lattice becomes tetragonal belonging to the *P*4_1_2_1_2 space group type, and the resulting superstructure can be determined by X-ray diffraction. In the case that the vacancy distribution is disordered, a cubic structure belonging to the *P*4_3_32 space group type results. Although maghemite is a ferric oxide, it cannot be synthesized *via* the direct precipitation of ferric ions but rather requires the oxidation of magnetite. For this reason, maghemite can be considered equivalent to fully oxidized magnetite. Although it has the same chemical formula as maghemite, hematite can be obtained through the precipitation of ferric ions. Unlike the two previous oxides, hematite belongs to the *R*3̄*c* space group type and comprises a hexagonal close-packed oxygen lattice in which two-thirds of the octahedral sites are occupied by ferric ions, within a rhombohedral lattice system belonging to the 3̄*m* trigonal crystal system. Hematite is the most stable iron oxide under ambient conditions.^7,15^

Several studies have examined the transformation of magnetite to maghemite or hematite (following eqn (1)), since the controlled oxidation (either partial or complete) of magnetite is essential to certain applications.^16^

1 4Fe_3_O_4_ + O_2_ → 6γFe_2_O_3_ → 6αFe_2_O_3_

The transition from magnetite to hematite is affected by redox conditions and occurs frequently in petrogenesis, because iron is the most abundant redox-sensitive element in most rocks. As an example, Zhao *et al.* reported that the transformation from magnetite to hematite under hydrothermal conditions depends on the solubility of ferrous species.^17^ The oxidation of magnetite in air is primarily affected by the ambient temperature,^18^ such that the oxidation kinetics increase along with temperature. At lower temperatures (<300 °C), the reaction rate is slow so that maghemite can exist as an intermediate over prolonged time spans. In contrast, at higher temperatures (>300 °C), the transition from maghemite to hematite occurs more rapidly. Thus, hematite is commonly found as an oxidation product at higher temperatures. Others researchers have determined that the oxidation rate of magnetite in air also depends on the particle size,^19^ purity^20,21^ and crystal morphology of the initial magnetite.^22–24^ Sidhu *et al.*^20^ studied the formation of maghemite in air at 200 °C and found no changes in morphology. They suggested that the transformation proceeded *via* the outward diffusion of ferrous ions and constructed a quantitative model for this process.

However, the majority of prior studies such as these were conducted using solid samples in air or pure oxygen, not under hydrothermal conditions, even though such conditions are commonly encountered in numerous geochemical formations and industrial processes.^25^ Among those, Yu *et al.*^2^ investigated the transformation of amorphous iron oxides in bacterial biofilms to hematite under hydrothermal conditions at 200 °C. Different transformation efficiencies, crystallinities and morphologies were found as the pH was varied from 1 to 10, and the optimal pH for the rapid production of well crystallized hematite was found to be 1. Swaddle and Oltmann^26^ compared the oxidation of magnetite in air and in water, both at 180 °C, and found that oxidation in air was completed within 15 min, whereas the reaction in water required several hours. Blesa and Matijević^27^ attributed these results to the blockage of cationic vacancies by abundant protons in the reaction solution. But the temperature effect on reaction kinetics was not studied. Tang *et al.*^28^ systematically investigated the oxidation of magnetite under hydrothermal conditions between 24 and 80 °C and concluded that the kinetics model devised by Sidhu *et al.*^20^ (based on the outward diffusion of ferrous ions) also applies to these conditions. At higher temperatures, Zhao *et al.*^17^ has investigated the transformation of magnetite to hematite between 140 °C and 200 °C. These experiments were conducted in the presence of NaCl in solution which was proved to increase the reaction kinetics making it difficult to be compared with most of the studies using diluted solutions. Only one set of experiments at 200 °C was conducted without NaCl in solution.

This literature review shows that both mechanism and kinetics of magnetite oxidation in hydrothermal conditions are still challenging. Thus, the application limits of the diffusion-based model proposed by Sidhu *et al.*^20^ are not defined and further experiments are needed to determine the mechanisms and to model the kinetics at high temperature.

A better description of the hydrothermal process is important for the nuclear industry, which motivated this study. Indeed, in nuclear power plants with pressurized water reactors (PWRs), the corrosion of carbon steel generates primarily magnetite in the secondary water vapour circuit due to the reducing conditions in this region.^26^ Given that the steam generators (SGs) in such plants transform water into vapour, the corrosion products become concentrated in the circuit, which may eventually lead to fouling and clogging.^27–30^ For this reason, deposited corrosion products must be periodically removed to ensure the integrity and heat exchange capacity of the SGs.^28,31^ In theory, hematite (the most stable iron oxide) can result from the oxidation of magnetite, and the formation of hematite could increase the risk of stress corrosion cracking (SCC) appearing in SG tubes.^32^ However, hematite also helps reducing the rate of flow-accelerated corrosion (FAC) because it is denser and less soluble compared with magnetite. Even if reducing conditions are maintained in the secondary circuit, a better knowledge of iron oxide speciation could improve our understanding of the numerous phenomena that occur in such circuits.^33^ Hence, the optimization of secondary circuit operation relies partially on obtaining more detailed information regarding the kinetics of magnetite oxidation under alkaline and low solute hydrothermal conditions from 25 °C to 275 °C, which are not covered by the available published studies.

The present study therefore aimed to provide a better understanding of the mechanism and kinetics of this reaction in the specific conditions of nuclear power industry. To this end, a series of experiments was conducted at four temperatures ranging from 120 to 275 °C with different reaction durations so as to assess the reaction kinetics, and a kinetics model was proposed.

## Experimental methods

### Autoclaves

Magnetite oxidation experiments were conducted in two autoclaves made of pure Ti alloy (grade 3). One was obtained from Parr and had an internal volume of approximately 400 mL while the other was obtained from Top Industrie (referred to hereafter as Top) and had an internal volume of about 450 mL. The Parr autoclave was sealed with a PTFE gasket while the Top autoclave was sealed using a graphite-coated Ti alloy. Each autoclave was equipped with a thermocouple inserted into the base of the reactor, a mechanical stirrer made of pure Ti alloy (grade 3) driven by an external motor and a sampling line.

### Materials

The reagents used in this study consisted of Milli-Q water (Millipore, specific resistivity of 18.2 MΩ cm at 25 °C), an ammonium hydroxide solution (Merck, Suprapur, 25%) and air (containing 21% oxygen). A 0.13 mmol L^−1^ ammonium hydroxide solution was prepared using Milli-Q water and the concentrated ammonium hydroxide solution. This solution had a pH of approximately 9.5 at 25 °C, which was equivalent to that in a PWR secondary circuit solution so as to mimic actual reaction conditions.

**Fig. 2:**
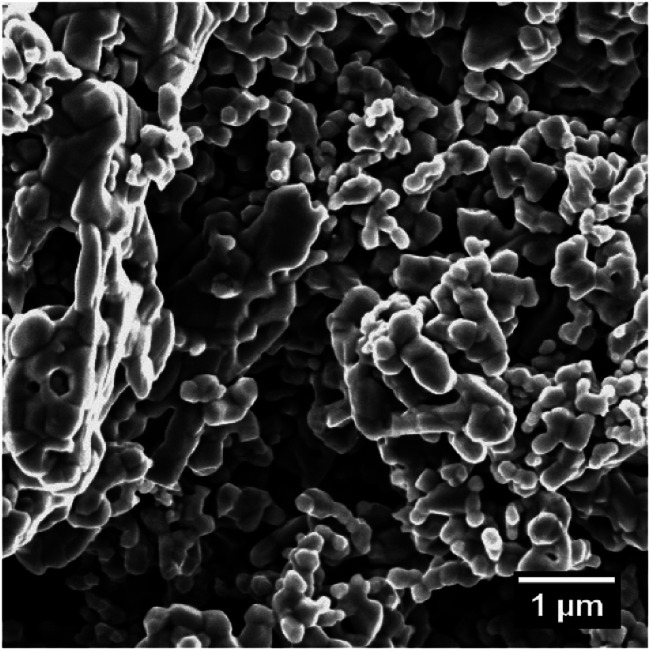
An SEM image of the commercial magnetite used in this study.

The synthetic magnetite used in this study was purchased from Alfa Aesar (catalogue code: 12962; purity: 99.997%) and was characterized before being used in oxidation experiments. Both X-ray diffraction (XRD) and titration analyses showed that this material had the requisite purity (97% according to titration), with a minor amount of hematite as an impurity (according to the XRD data). Scanning electron microscopy (SEM) images indicated that this magnetite was composed of agglomerations of particles several hundreds of nanometres in size (as shown in Fig. 2). Another batch of the same commercial magnetite was previously characterized using complementary techniques^36^ and the particle sizes were determined to be in the range of 100 and 1000 nm using laser diffraction spectroscopy (Malvern Mastersizer 2000, laser wavelengths at 466 nm and 633 nm). The specific surface area of this material was assessed based on the Brunauer–Emmett–Teller (BET) method, employing krypton gas, and was found to be 1.7 m^2^ g^−1^. This corresponds to a 690 nm particle size assuming a spherical morphology and a density of 5.18 g cm^−3^ as reported by Cornell *et al.*^15^

### Experiments

Each experiment was performed using a specific reaction temperature and time. The required volume of the ammonium hydroxide solution was mixed with a quantity of the synthetic magnetite and the mixture was introduced into the autoclave, which was then sealed and heated to the reaction temperature. The volume in the autoclave above the solution was filled with air at atmospheric pressure (1 bar). The point in time at which the solution reached the desired reaction temperature was considered to represent *t* = 0. The pressure in each autoclave was maintained at the saturation pressure of water at the reaction temperature. The temperature was maintained at a constant value throughout the reaction time and then decreased to room temperature after heating was stopped. Solid samples were recovered through filtration and were dried under vacuum at ambient temperature. The pH of the supernatant liquid was also measured. The experimental conditions are summarized in Table 1.

**Table 1:** Summary of experimental conditions

Samples	Reaction temperature (°C)	Reaction pressure (bar)	Autoclave type	Magnetite loading (g)	Ammonia solution (g)	Volume of air (mL)	Reaction time (h)
120-1	120	2	Parr	0.5	100	300	24
120-2	120	2	Parr	0.5	100	300	66
120-3	120	2	Parr	0.5	100	300	115
150-1	150	4.8	Top	0.56	112	338	4
150-2	150	4.8	Parr	0.5	100	300	48
180-1	180	10	Parr	0.5	100	300	4
180-2	180	10	Parr	0.5	100	300	16
180-3	180	10	Parr	0.5	100	300	65
275-1	275	59.5	Top	0.56	112	338	4
275-2	275	59.5	Parr	0.5	100	300	16
275-3	275	59.5	Parr	0.5	100	300	24

An excess of air relative to the magnetite loading was introduced into the autoclave to ensure a sufficient oxygen supply and to limit variations in the oxygen concentration in the solution due to the consumption of oxygen during the oxidation of Fe(ii) to Fe(iii). Reagent loadings were varied in proportion to the volume of the autoclave.

### Sample analysis

After recovery, solid samples were stored under nitrogen to avoid undesired oxidation. Crystallographic analysis of these samples was performed using powder XRD, employing a Bruker D8 Endeavor diffractometer. Phase analysis and semi-quantification of each phase were carried out with the X'pert HighScore software package and, while searching for matching phases, the elements were restricted to Fe and O. Semi-quantification was performed based on the reference intensity ratio (RIR) analysis integrated into the X'pert Highscore package. Solid samples were also digested in a hydrochloric acid solution (6 mol L^−1^) under nitrogen until completely dissolved, then assessed by titration. A classic spectrophotometric analysis with 1,10-phenanthroline was adapted to determine the Fe(ii) to Fe ratio (Fe(ii)/Fe) in these solid samples. Prior to this analysis, samples were digested in an acetate buffer solution containing 1,10-phenanthroline and the absorbance of this solution at 510 nm was determined using a Cary 100 Scan UV-visible spectrophotometer. This value was compared with the absorbance of the same solution following the addition of diethylhydroxylamine as a reducing agent. A more detailed description of this method can be found in the (ESI†). These samples were also assessed by SEM using a Quanta FEI 650F instrument operating in the secondary electron mode. TEM and electron diffraction analysis have been carried out using a FEI OSIRIS microscope working at 200 kV located at EDF Lab Renardières, France.

## Results and analysis

### Oxidation kinetics

The magnetite contents of the oxidized samples were plotted against reaction time to allow a study of the oxidation kinetics. The magnetite concentration in each specimen was calculated based on the Fe(ii)/Fe ratio obtained from titration, assuming that Fe(ii) was present only in magnetite.

**Fig. 3:**
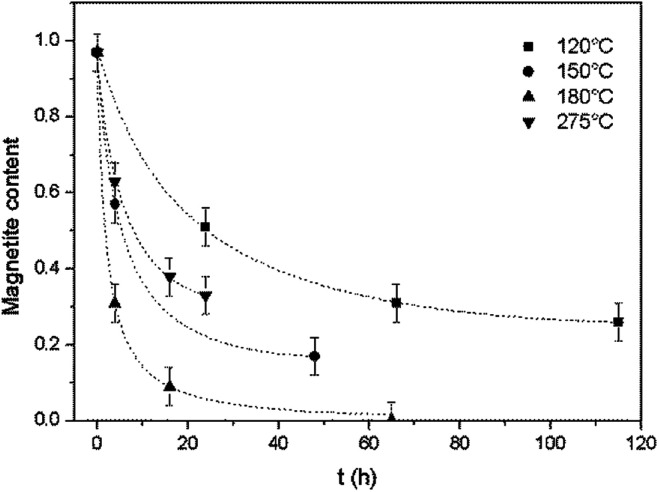
Oxidation reaction kinetics of magnetite under hydrothermal conditions based on reaction with dissolved oxygen at various temperatures, together with trend lines.

As shown in Fig. 3, for all reaction temperatures tested, the oxidation kinetics presented the same characteristics. During the early stage of oxidation, the reaction rate was high but then decreased as the reaction continued, thus exhibiting a parabolic trend. The effect of temperature on the reaction kinetics was also assessed. From 120 to 180 °C, the reaction kinetics increased with increasing temperature, in agreement with the results of a previous study that showed that the kinetics of the transformation from magnetite to hematite increased on going from 140 to 200 °C.^17^ However, at 275 °C, the reaction kinetics slowed compared with the results at 180 °C. This outcome indicates the possible existence of a transition temperature between 180 and 275 °C, and is discussed in greater detail below.

### Phase analysis and morphology

All samples were found to be composed of either a single phase or several phases, including magnetite (cubic), maghemite (cubic), maghemite (tetragonal) and hematite (trigonal). Circle charts showing the phase composition (in mass fractions) were generated based on semi-quantification by XRD. The results for the samples produced at 150 and 180 °C are not presented here because these specimens were very similar to the 120 °C sample. Detailed data can be found in the ESI† for this paper.

At 120 and 150 °C, the maghemite was found to have a cubic structure, indicating a disordered vacancy distribution. In contrast, the maghemite in the samples presented both cubic and tetragonal structures at 180 °C. Therefore, the formation of an ordered vacancy distribution in the maghemite evidently required a minimum energy input and so was possible only at high temperatures. At low temperatures only a disordered vacancy distribution was obtained.

**Fig. 4:**
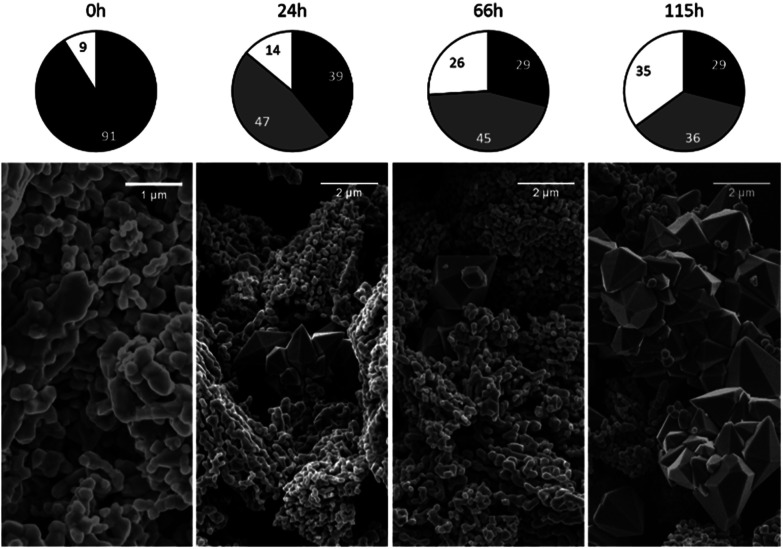
Crystalline phase compositions and SEM images of 120 °C samples after various time spans (chart legend: black = magnetite, grey = maghemite, white = hematite).

**Fig. 5:**
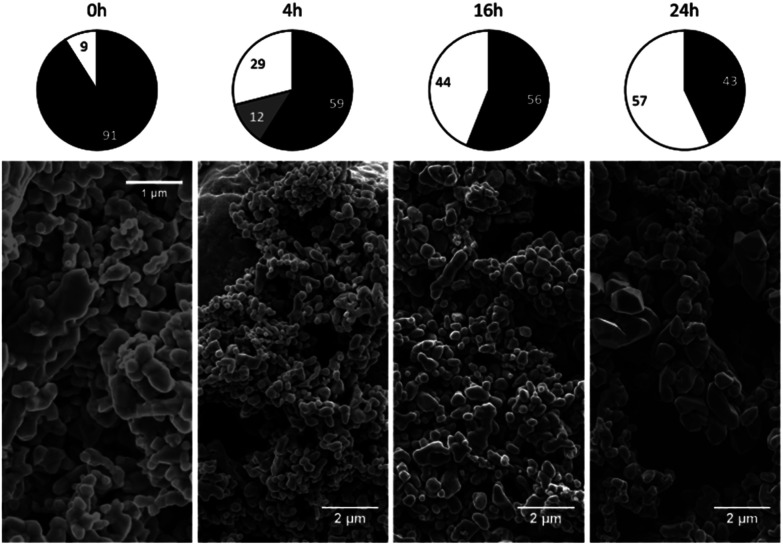
Crystalline phase compositions and SEM images of 275 °C samples after various time spans (chart legend: black = magnetite, grey = maghemite, white = hematite).

**Fig. 6:**
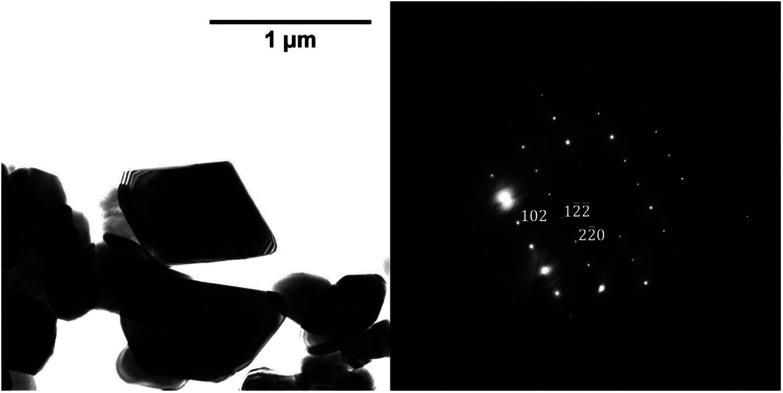
TEM image of a hexagonal bipyramid crystal and power spectrum of image which serves to identify the crystal, zone axis [221̄].

The phase composition data and morphologies (as shown by SEM images) of the corresponding samples are presented in Fig. 4 and 5. It can be seen that oxidation between 120 and 180 °C leads to the formation of new particles with morphologies clearly different from that of commercial magnetite. The majority of the new particles are hexagonal bipyramidal while others are rhombohedral. To the best of our knowledge, magnetite and maghemite have never been reported with hexagonal bipyramidal or rhombohedral morphology as expected from the cubic or tetragonal crystal system. In addition, maghemite generated by a solid-state transformation typically preserves the morphology of the precursor material.^15^ Hence, maghemite formed *via* the oxidation of magnetite will tend to adopt the morphology of the magnetite. Consequently, both the magnetite and maghemite preserved the initial morphology of the commercial magnetite and appear as agglomerations of rounded particles (as shown in Fig. 2). In the sample 180-3, the hexagonal bipyramids and rhombohedra can only be hematite since the sample consists of 100% hematite according to XRD. In another sample 180-0 (blank test in the same conditions as 180-3 with air replaced by nitrogen gas), the morphology did not change regardless of the 16 hours of hydrothermal treatment of magnetite. Besides, hexagonal bipyramids and rhombohedra are typical hematite morphologies, especially rhombohedra which is commonly reported for this material.^15,37–39^ Therefore those hexagonal bipyramids and rhombohedra are thought to be hematite. Confirmation has been made using electron diffraction under TEM. A hexagonal bipyramid particle has been clearly identified as hematite from diffraction spots (as shown in Fig. 6). A dodecahedral hexagonal bipyramidal morphology was previously obtained with the aid of F^−^ (>24 mM) in a hydrothermal solution by Lv *et al.*^40^ However, an analysis of our solutions indicated a F^−^ concentration below 1 mM, suggesting that other anions might also have promoted the formation of this morphology.

Following the oxidation reaction at 275 °C, the SEM images show no evidence for the characteristic morphology of hematite, even when over half the magnetite has been oxidized to hematite. This indicates that the oxidation process at this temperature was different from that which occurred between 120 and 180 °C.

### Effect of stirring speed

The Parr autoclave, unlike the Top unit, was not equipped with a means of measuring the rotational rate of the stirring mechanism. The effects of the rotational rate on the reaction kinetics were assessed by performing experiments under the same conditions as those used for run 150-1 (as shown in Table 2) but with different rotational speeds. The stirring conditions and results of these experiments are provided in Table 2.

**Table 2:** The effect of stirring speed on magnetite oxidation

Run number	150-1	150-3	150-4
Stirring speed (rpm)	100	200	400
Final magnetite content	57%	57%	57%

These results confirm that the stirring speed had no effect on the reaction kinetics in the range examined in this study. Therefore, it is likely that the slight difference in the rotational rates of the two autoclaves did not induce any variation in the magnetite oxidation process. These results also indicate that the transport of DO is not a rate limiting factor for this reaction under the conditions being employed.

### pH decrease

The pH of the reaction solution was measured before and after each trial. The initial pH was adjusted at room temperature to approximately 9.5 using ammonium hydroxide but the solution pH typically dropped to the acidic range during the experiment, with final pH values as low as 4. Part of this drop was due to the evaporation of ammonia, although this would only be expected to produce a neutral pH.

The autoclaves were cleaned before each test. However impurities may still be present on the inner surface of autoclaves. These impurities can be easily released into solution especially at high temperature during experiments, causing pH shift. Additionally, dissolution of CO_2_ in solution acidifies this latter. But during blank trials, the pH fell only to approximately 5 due to the release of impurities and dissolution of CO_2_. Thus another phenomenon was responsible for the lower final pH values.

**Fig. 7:**
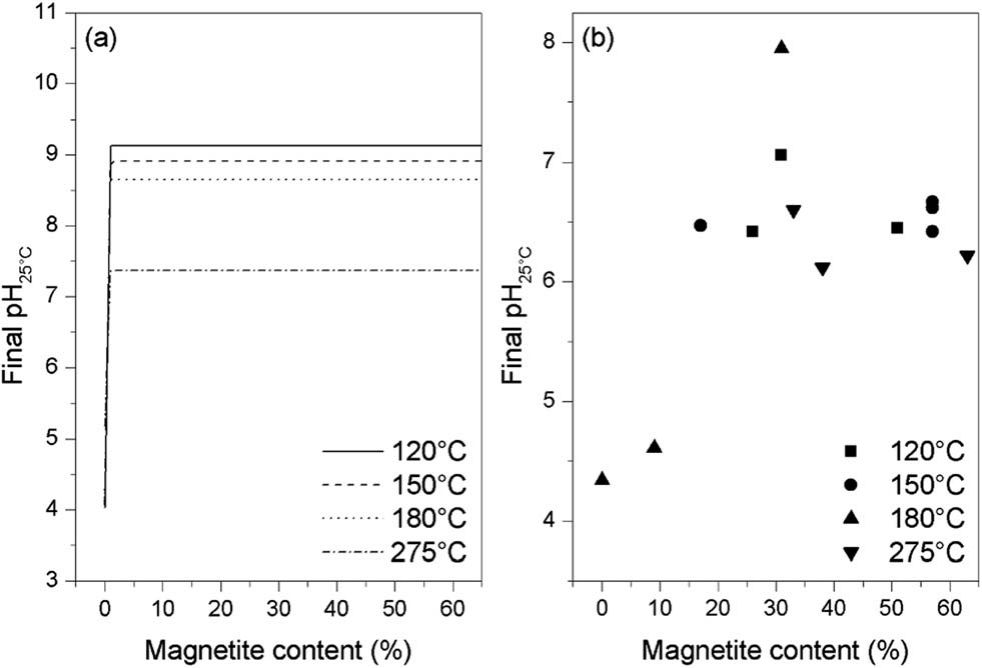
Final pH values from thermodynamic calculations (a) and from experiments (b) for various magnetite concentrations in samples at the end of reaction.

Thermodynamic calculations were carried out using the Phreeqc program and the Lawrence Livermore National Laboratory llnl.dat database. Applying the same conditions as used in the experiments and adjusting the amount of oxygen, different final states of the solution were simulated. It was found that, when the magnetite is partially oxidized, the solution pH is lower than the initial pH but remains stable and independent of the magnetite content (as shown in Fig. 7a). However, upon complete oxidation of the magnetite, the pH drops to around 4 or 5 depending on the temperature. Similar results were obtained for all four temperatures used in this study, demonstrating that the acidic pH only appears at the end of or after the oxidation of the magnetite. By plotting the final pH_25 °C_ value against the magnetite content of the oxides resulting from these trials (as shown in Fig. 7b), it was found that the pH stays stable and near neutral when more than 10% magnetite remains in the final oxide, and drops to between 4 and 5 only when the magnetite content is lower than 10%. These results are consistent with the thermodynamic calculations, indicating that the pH decrease might be the result of a systematic side reaction rather than a random error.

The detailed thermodynamic calculations suggest that the oxidation of ammonia is responsible for the pH drop at the end of the reaction. Following the complete oxidation of the magnetite to hematite, excess oxygen could oxidize the ammonia to produce nitrate anions while also generating protons, as in eqn (2), to lower the pH. In fact, the oxidation of the small remaining quantity of magnetite at the end of the reaction process (that is, <10% magnetite) could become increasingly difficult. Therefore, the oxidation of ammonia could begin even at this point, in contrast to the results of the thermodynamic calculations, which suggest that ammonia starts to be oxidized only after the total oxidation of the magnetite.

2 2O_2_(aq) + NH_3_(aq) = NO^−^_3_ + H_3_O^+^

**Fig. 8:**
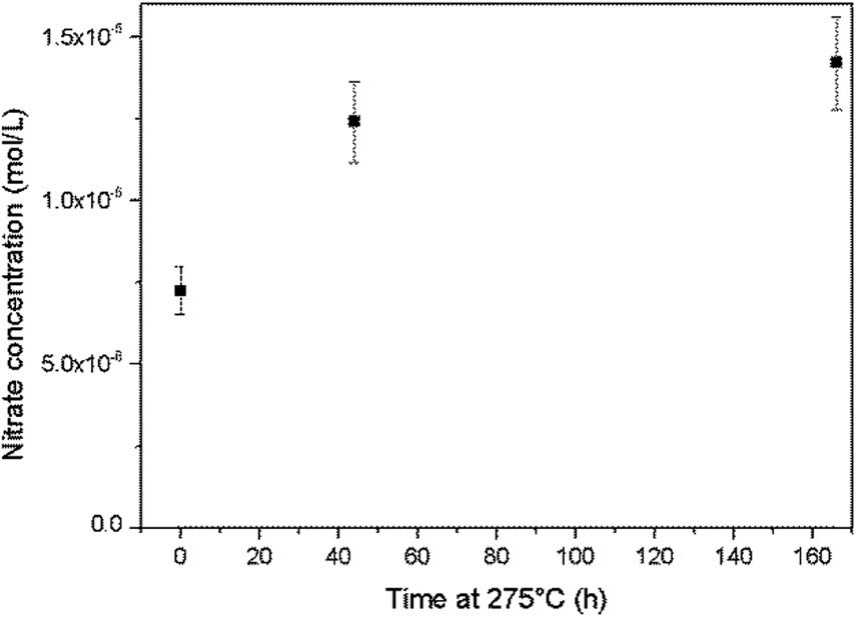
Increases in the nitrate concentration during autoclave cleaning with an ammonium hydroxide solution at 275 °C.

An alkaline cleaning process using a 0.66 mmol L^−1^ ammonium hydroxide solution at 275 °C was applied to the Top autoclave and aliquots of the wash solution were periodically analysed. The nitrate concentration in the solution was found to increase during this process (Fig. 8), which provides evidence for ammonia oxidation.

It is also worth noting that the *ex situ* analysis of samples taken from the hydrothermal system may cause erroneous results. During the cooling process, significant changes in the temperature and pressure of the sample over a short period of time may lead to phenomena such as oxidation, variations in aqueous speciation or the formation of colloids.^25,41^ Hence, the results of *ex situ* analysis should be treated with caution. Assuming that the analytical data are reliable, because the pH remained stable and near neutral during the oxidation reaction and dropped to approximately 4 only at the end of the reaction, this phenomenon is not considered as an issue for this study.

## Mechanism and kinetics model

### Reaction mechanism

Based on our observations, a reaction mechanism can be proposed. Because different behaviours were observed above and below 180 °C, this discussion will be separated into two parts dealing with these two temperature regions.

#### Between 120 and 180 °C

Maghemite was identified as one of the oxidation products at these temperatures. For all reaction temperatures in the range from 120 to 180 °C, the maghemite content increased rapidly during the early stage of the reaction and then remained constant or decreased afterwards. In contrast, the magnetite content decreased monotonically and the hematite content increased monotonically. Thus, the maghemite was very likely an intermediate during the transformation from magnetite to hematite.

**Fig. 9:**
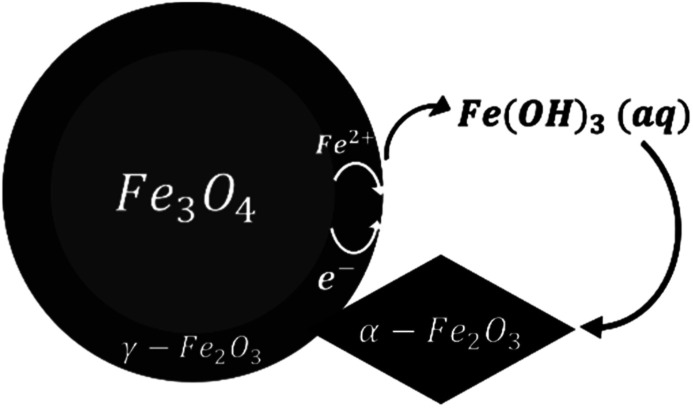
A schematic of the oxidation reaction mechanism between 120 and 180 °C.

Both magnetite and maghemite present similar crystal structures, and so the transformation between these two phases can take place without significant structural changes. Some prior studies have reported that the transformation between magnetite and maghemite proceeds *via* the solid state diffusion of ions. This process could involve the inward diffusion of oxygen or, as is more commonly accepted, the outward diffusion of ferrous ions to produce a Fe_3−*x*_O_4_ solid solution or a core/shell structure made of magnetite/maghemite^19,34,42^ with the oxidation of ferrous ions at the particle surfaces by O_2_.^43^ Iyengar *et al.*^1^ recently provided direct evidence for this core/shell structure using techniques such as transmission electron microscopy. Because the DO in the reaction solution would be in direct contact with the magnetite surface, the initial oxidation process is believed to occur at this surface to form a maghemite layer. In this mechanism, ferrous ions diffusing out of the magnetite dissolve in the surrounding solution and are subsequently oxidized by DO to produce aqueous ferric species. In the present trials, the primary species would be Fe(OH)_3_(aq) based on thermodynamic predictions. The solubility of such species was reported to be an important factor in the transition from magnetite to hematite under similar hydrothermal conditions.^17^ Simultaneously, maghemite dissolves in the solution to also produce Fe(OH)_3_(aq) which then reprecipitates to form hematite, resulting in the coexistence of three phases. This mechanism is illustrated in Fig. 9.

This hypothesis is supported by SEM observations. In the case of samples containing magnetite, maghemite and hematite, the majority of crystals retained the morphology of the original commercial magnetite. Other crystals clearly had different morphologies, very likely corresponding to hematite according to reports regarding the morphologies of such phases.^15,37–40,44^ Apart from the crystals appearing to be hematite, the remaining particles presented only one type of morphology, demonstrating the direct transformation from magnetite to maghemite without morphological change.

The oxidation of magnetite to maghemite through the formation of ferrous ions is a diffusion-based process and thus can be described using a spherical diffusion rate law with diffusion of the ions as the rate limiting step for magnetite consumption. The formation of hematite involves two steps, dissolution and reprecipitation, and the first of these steps proceeds *via* two mechanisms: the dissolutions of ferrous ions and of maghemite. Since maghemite can exist for prolonged time spans under these conditions and ferrous ions are generally more soluble than ferric ions, the dissolution of maghemite is most likely the rate limiting step. In this case, because the ferric species would be present at a low concentration in the reaction solution, precipitation to form hematite likely proceeds based on a small number of nuclei that slowly grow into large crystals. This is in agreement with the SEM images shown in Fig. 4.

#### At 275 °C

**Fig. 10:**
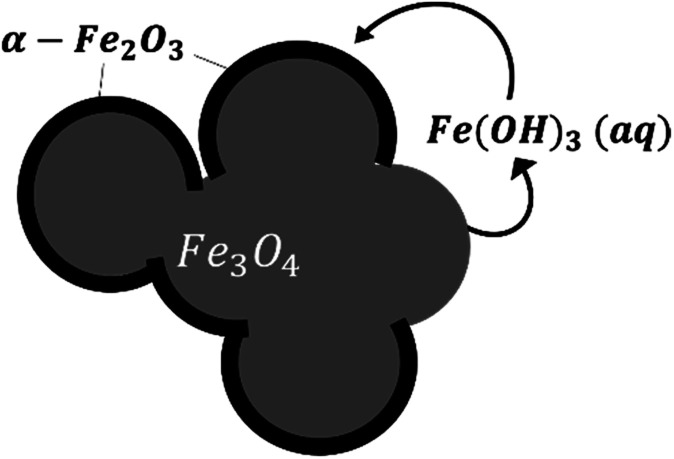
A schematic of the oxidation reaction mechanism at 275 °C.

Maghemite exists only during the very early stage of the reaction and its amount is greatly reduced compared with that at lower temperatures. This indicates that the diffusion process could be impeded and the dissolution of maghemite could be promoted. Furthermore, no hematite with bipyramidal or rhombohedral morphologies was observed as at the lower temperatures even though considerable amounts of hematite were identified at 275 °C. This could have been the result of much faster growth of hematite on the magnetite surface, leading to the formation of a core/shell structure and preservation of the initial morphology of the commercial magnetite (as illustrated in Fig. 10). In this scenario, the diffusion of ferrous ions or oxygen through the hematite is unlikely due to its compact structure, and thus transformation can only occur at the uncovered magnetite surface. This decrease in the surface area available for transformation produces a decrease in reaction kinetics. Moreover, the maghemite does not persist under these conditions, indicating that it dissolves rapidly. Consequently, maghemite dissolution is no longer the rate limiting step. The magnetite would be expected to dissolve *via* the uncovered surfaces and to subsequently oxidize to give aqueous ferric species that then precipitate to form hematite at the magnetite surface. The SEM images in Fig. 5 indicate a slight increase in particle size over time. Hence, at this temperature, the reaction probably proceeds through diffusion and maghemite formation during the early stage, following by the growth of hematite directly from magnetite on the surface, which slows down the reaction kinetics.

### Kinetics model

Several kinetics models were evaluated to determine the limiting step, and the diffusion-controlled model was determined to be optimal. This model is based on spherical diffusion and was first applied to the study of the oxidation of magnetite in air by Sidhu *et al.*^20^ This model was subsequently applied to magnetite oxidation under a controlled atmosphere by Bourgeois *et al.*^16^ and has also been used to assess the aqueous oxidation of magnetite at low temperatures (≤80 °C) by Tang *et al.*^23^ All of these studies showed good agreement between the experimental data and the model predictions, and obtained similar values for the constants in the model (the activation energy and pre-exponential factor for the diffusion coefficient). In these studies, a simplified form of this model intended for short reaction times was used. However, short reaction times cannot necessarily be assumed in our study, in which larger amounts of oxidized magnetite were obtained. Hence, a more general form^45^ of this model was used, as discussed below.

This diffusion model can be described by the following eqn (3).3
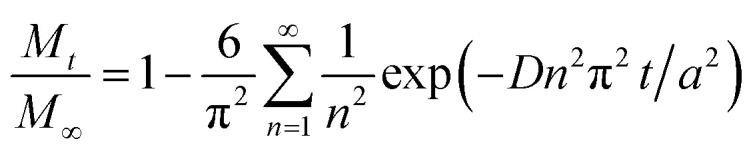
Here, *M*_*t*_ and *M*_∞_ are the quantities of ions that have diffused to the outer surface at time *t* and at infinite time, respectively, *D* is the diffusion coefficient and *a* is the spherical radius. One assumption associated with this model is that the DO level is in excess and so does not limit the reaction rate. This is in agreement with the finding that the stirring speed (and so the transport of DO in the solution) does not have any effect on the reaction rate. Hence, the concentration of DO does not appear in this model.

There are several parameters that play important roles in this model, and the choice of the spherical radius has a direct impact on the reaction kinetics. Although the actual morphology of the magnetite can be seen in Fig. 2, the particles were considered to be perfectly spherical and their size was calculated from the BET specific surface area data. Another important parameter is the diffusion coefficient, which is thought to follow the Arrhenius law as in eqn (4).

4 *D* = *D*_0_ exp(−*E*/*RT*)

Here, *D*_0_ is the pre-exponential factor, *E* is the activation energy, *R* is the ideal gas constant and *T* is the temperature in degrees Kelvin. Both the pre-exponential factor and activation energy were obtained from experimental data in the literature, which were found to be relatively constant. The values reported by Tang *et al.*^35^ (*E* = 21.0 kcal mol^−1^ and *D*_0_ = 7.2 × 10^−5^ cm^2^ s^−1^) were used, since these were also obtained under hydrothermal conditions.

**Fig. 11:**
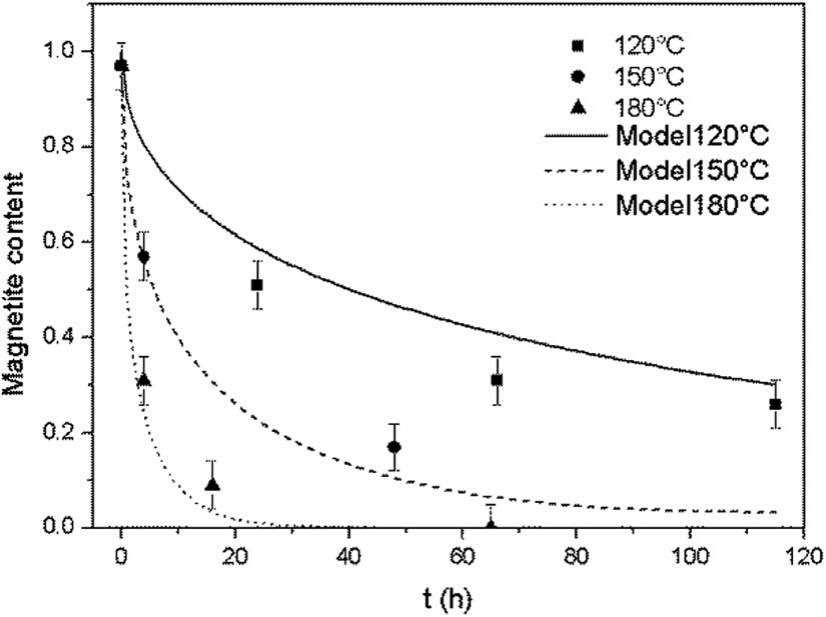
Comparison of the experimental magnetite oxidation data to that generated using a diffusion model at various temperatures.

The model was used to generate simulated kinetics data based on the above parameters, and these are compared with experimental data in Fig. 11 without fitting. Note that this is an isothermal model that applies only to constant temperature conditions whereas, in our experiments, there was always a transient phase during which the temperature increased from ambient to the reaction temperature, as well as a cooling phase. These steps were incorporated into the calculations by considering each transient phase as a series of several constant phases with different temperatures and durations. The temperature in each of these steps was set equal to the average temperature during the phase. The results of numerical calculations demonstrated that the initial temperature increase step had an important effect on the kinetics, while the effect of the cooling stage was negligible. This difference is due to the rapid diffusion during the initial stage and slower diffusion during the last stage.

At temperatures ranging from 120 to 180 °C, the diffusion-controlled model accurately simulated the oxidation reaction (as shown in Fig. 11), indicating that the diffusion of iron ions in the magnetite/maghemite was the rate limiting step in this temperature range. However, at higher temperatures, the model predicted increased kinetics that did not agree with the experimental observations. As discussed in the previous section, the reaction mechanism changed between 180 and 275 °C as a layer of hematite was formed on the surface of the magnetite. Being denser than magnetite, the hematite layer impedes the diffusion of iron and oxygen, hence slowing the reaction.

## Conclusions

This study has provided new information regarding the mechanism and kinetics of magnetite oxidation under high temperature hydrothermal conditions. A change in the reaction mechanism with increasing temperature was observed for the first time. Two different reaction mechanisms were evident. Between 120 and 180 °C, the formation of maghemite through the diffusion of ferrous ions occurs along with the dissolution of maghemite and precipitation of hematite. At 275 °C, the hematite is directly formed on the magnetite surface. The applicable temperature range of the associated diffusion-controlled model has been extended to 180 °C compared with the value of 80 °C used in previous studies. A simple analytical method based on spectrophotometry for the determination of the iron(ii) proportion in the iron oxide has also been developed during this study, and shows good quantitative results.

The data demonstrate that the magnetite oxidation kinetics do not always increase with temperature. Various competing mechanisms might be affected differently by changes in temperature, which could result in different dominant mechanisms at varying temperatures.

## Conflicts of interest

There are no conflicts to declare.

## Supplementary Material

RA-009-C9RA03234G-s001
