# Supplementary material for: Mechanism and kinetics of magnetite oxidation under hydrothermal conditions
Source: RSC Adv. 2019 Oct 18;9(58):33633–42. doi: 10.1039/c9ra03234g (PMC9073545; doi:10.1039/c9ra03234g)
Supplement: RA-009-C9RA03234G-s001 [file RA-009-C9RA03234G-s001.pdf]

Electronic Supplementary Material (ESI) for RSC Advances.
This journal is © The Royal Society of Chemistry 2019

Mechanism and kinetics of magnetite oxidation under hydrothermal conditions

Zimin Li,[ad] Corinne Chanéac,[b] Gilles Berger,[c] Sophie Delaunay,[d] Anaïs Graff, [d] and Grégory Lefèvre*[a]

Electronic supplementary information

Titration of Fe(II)/Fe ratio in iron oxides using spectrophotometric analysis

This method is based on the spectrophotometric measurement of Fe(II) concentration in a solution using orthophenanthroline. In theory, orthophenanthroline forms a complex with ferrous ions within the range of pH from 2 to 9 at ambient temperature[1]. Such solution presents a peak of absorbance of visible lights at the wave length of 510 nm and the absorbance is proportional with the concentration of the complex between orthophenanthroline and ferrous ions. By adding excessive orthophenanthroline to a solution of ferrous ions, the concentration of the latter can be obtained through spectrophotometric analysis.

In this study, this method has been adapted to analyze iron oxides. First of all, between 5 mg and 10 mg iron oxides are mineralized in 20 mL HCl of about 6 M until total dissolution. This process is carried out under nitrogen protection to avoid oxidation and under magnetic stirring to accelerate dissolution. Then the ferrous ions concentration is obtained using the method described previously. 1 mL solution of mineralization is mixed with 1 mL solution of orthophenanthroline (10 g/L) and 1 mL buffer solution (1 mol/L CH_3COOH and 1 mol/L CH_3COONa). The absorbance of this solution mixture at 510 nm is recorded when stable (about 5 min after mixture). This same solution is then added with excessive diethylhydroxylamine (DEHA) compared to iron to reduce all ferric ions to ferrous ions. In practice, 2 µL solution containing 10% DEHA is added. Since the volume is negligible compared to the volume of the Fe-containing solution, the change of volume due to addition of DEHA is not taken into account. When stable (about 30 min after addition of DEHA), a second spectrophotometric analysis is performed on this reduced solution and the iron concentration (sum of ferrous and ferric ions) is obtained. The ratio between the two absorbances obtained is the Fe(II)/Fe ratio in the analyzed iron oxides.

This method has been tested and validated using commercial magnetite (Alfa Aesar, 12962, 99.997%) as reference material. The Fe(II)/Fe ratio measured using this method was 0.327, whose value is 0.333 in theory. The uncertainty of this method has been estimated at 5%. By supposing that our samples are composed of Fe_3O_4 and Fe_2O_3, the molar percentage of each composition can be retrieved through the Fe(II)/Fe ratio using the following equations.

$$\%Fe_3O_4 = \frac{2Fe(II)/Fe}{1 - Fe(II)/Fe} \tag{1}$$

$$\%Fe_2O_3 = 1 - \%Fe_3O_4 \tag{2}$$

However this method presents still certain drawback. The absorbance at 510 nm is slightly affected by other factors like ferric ions. When the Fe(II)/Fe ratio is near zero, this effect becomes non negligible. Hence this method is not capable in analyzing hematite.

Table 1 – Fe(II)/Fe ratio of samples

Samples	Temperature	Reaction time	Fe(II)/Fe
120-1	120	24	0.203
120-2	120	66	0.134
120-3	120	115	0.114
150-1	150	4	0.222
150-2	150	48	0.078
180-1	180	4	0.136
180-2	180	16	0.041
180-3	180	65	0.047*
275-1	275	4	0.239
275-2	275	16	0.158
275-3	275	24	0.143

*Sample composed of pure hematite according to XRD. Thus magnetite content is considered to be 0.

Results detail

Crystalline phase composition of samples at 150°C and at 180°C were obtained in the same way as at 120°C and 275°C as presented in the article. Since they are similar to that at 120°C, they are presented hereafter as supplementary information.

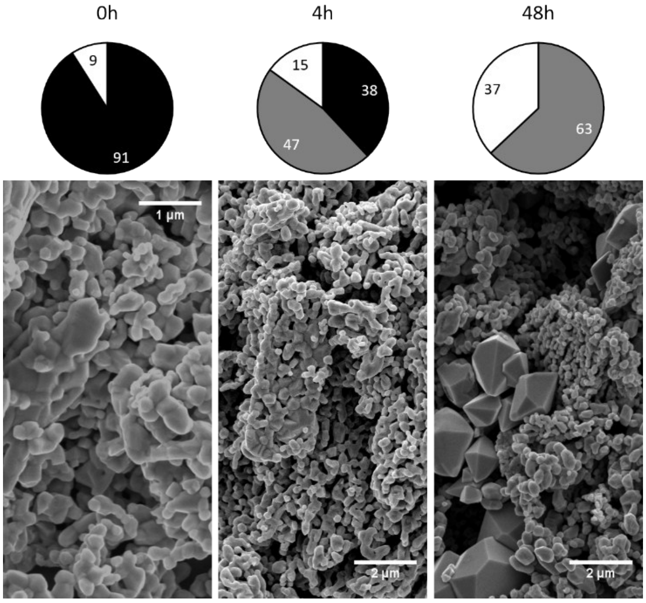

Figure 1 – Crystalline phase composition and SEM images of 150°C samples (in circle chart, black: magnetite, grey, maghemite, white: hematite)

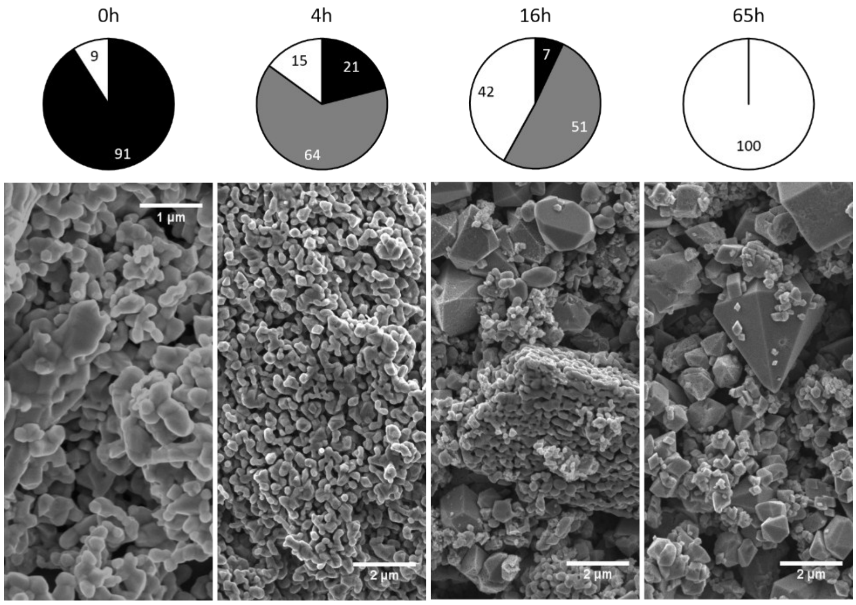

Figure 2 – Crystalline phase composition and SEM images of 180°C samples (in circle chart, black: magnetite, grey: maghemite, white: hematite)

X-ray spectra of all samples are presented hereafter. The diffractometer used is equipped with a cobalt source with $K_{\alpha1}$ = 1.789010 Å and $K_{\alpha2}$ = 1.792900 Å.

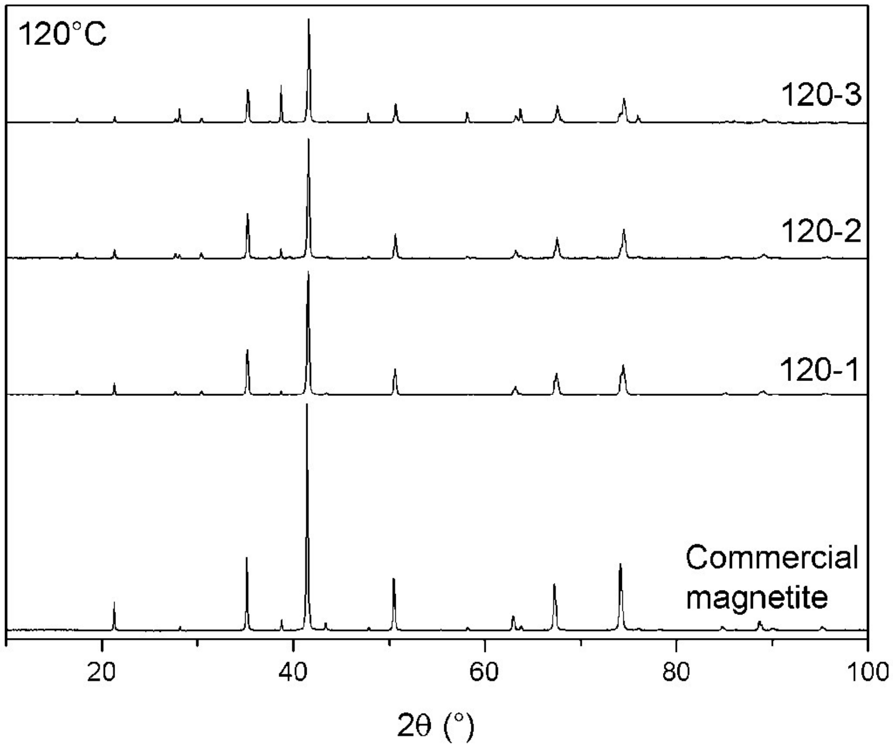

Figure 3 – XRD spectra of samples at 120°C compared to that of commercial magnetite

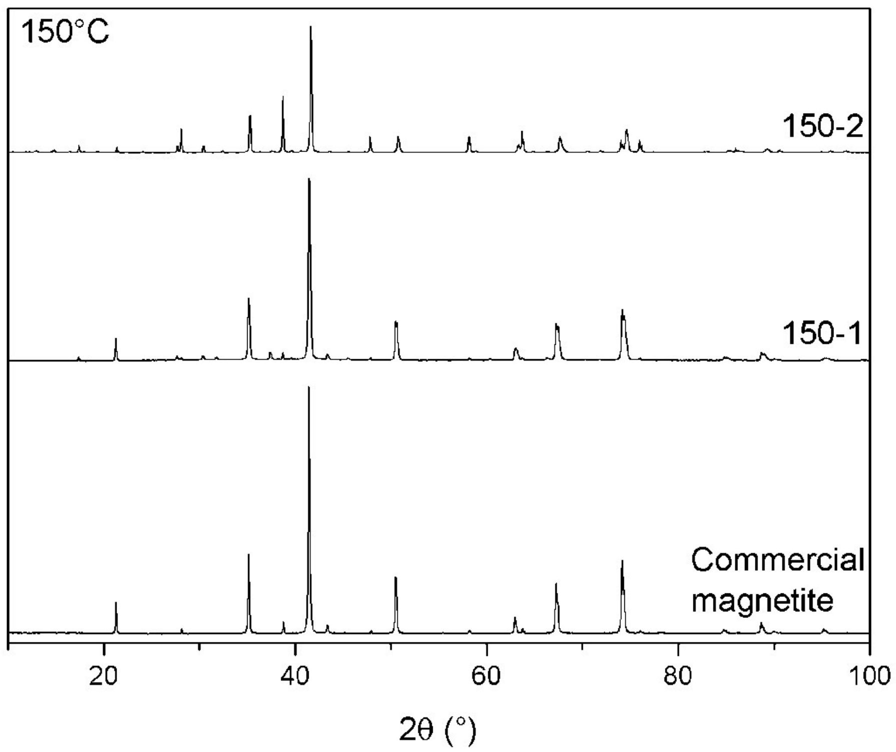

Figure 4 - XRD spectra of samples at 150°C compared to that of commercial magnetite

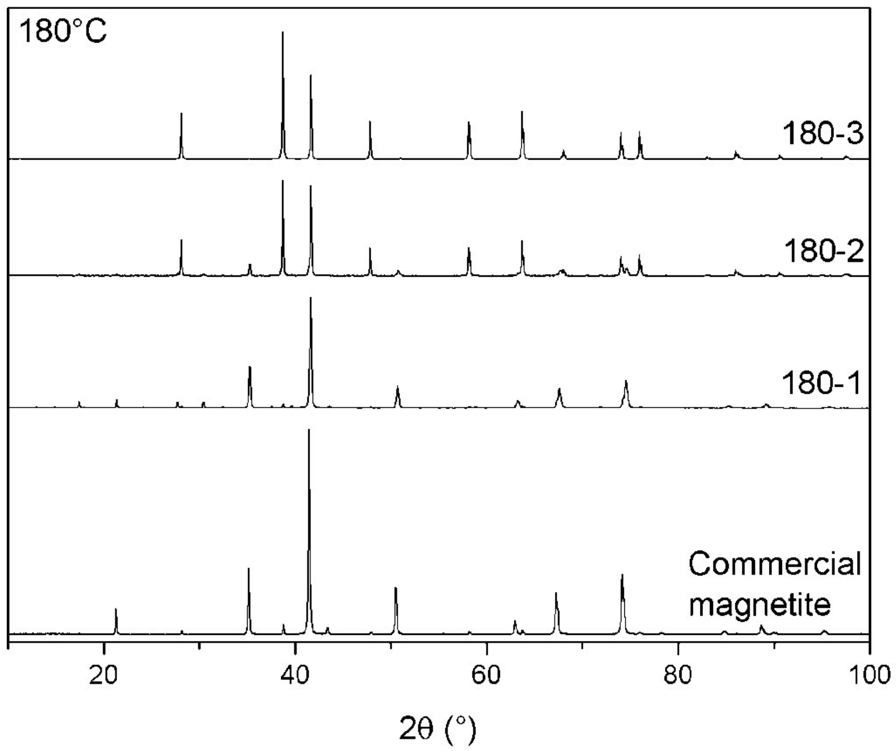

Figure 5 - XRD spectra of samples at 180°C compared to that of commercial magnetite

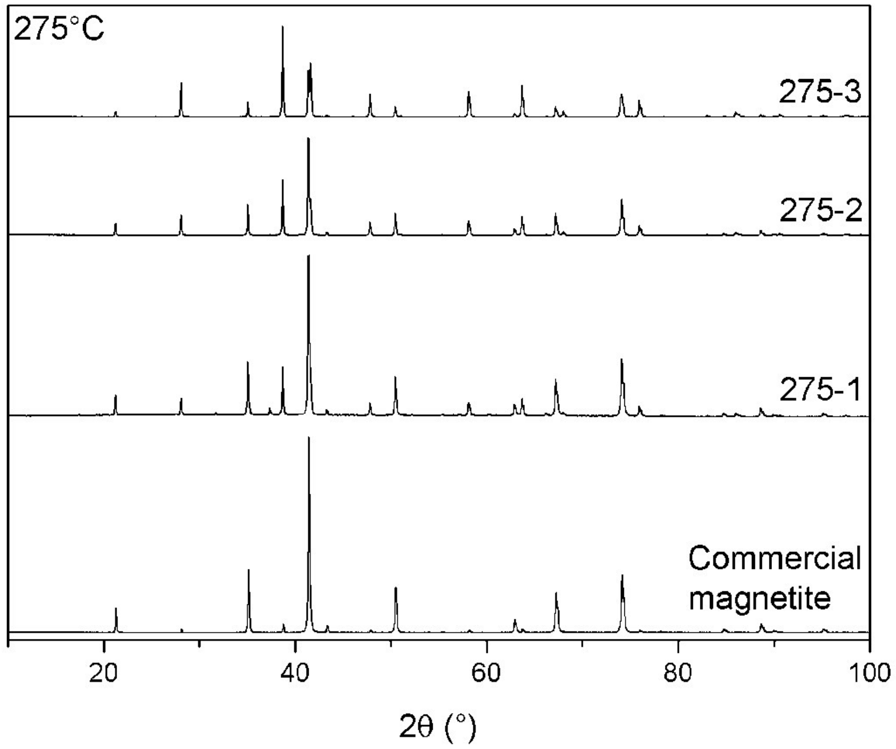

Figure 6 - XRD spectra of samples at 275°C compared to that of commercial magnetite

References:

[1] R. K. Adhikamsetty, N. R. Gollapalli, and S. B. Jonnalagadda, "Complexation kinetics of Fe^{2+} with 1,10-phenanthroline forming ferroin in acidic solutions," *International Journal of Chemical Kinetics*, vol. 40, no. 8, pp. 515–523, Aug. 2008.
